# Is psychopathy a disorder or an adaptation?

**DOI:** 10.3389/fpsyg.2012.00549

**Published:** 2012-12-18

**Authors:** Liane J. Leedom, Linda Hartoonian Almas

**Affiliations:** ^1^University of BridgeportBridgeport, CT, USA; ^2^Aftermath: Surviving Psychopathy FoundationYorkville, IL, USA

In a recent article in Frontiers in Evolutionary Psychology, Krupp et al. (2012) propose that (1) psychopathy is associated with nepotism and therefore (2) psychopathy is not a disorder but an evolutionarily based life strategy. In this commentary, we will address these two points, and outline an alternative evolutionary theory.

## Is psychopathy associated with nepotism?

Krupp et al. (2012) state, “individuals executing well-designed strategies, a necessary feature of psychological adaptations, should tend to be nepotistic—providing aid to close genealogical kin and/or sparing them from harm (p. 2).” They then present data linking psychopathy to index offenses involving violence toward non-relatives as opposed to relatives. Total Psychopathy Checklist-Revised (PCL-R) scores were positively correlated with victimization of non-relatives. These findings agree with prior studies linking psychopathy to violent victimization (Coid and Yang, 2011). The idea that psychopathic individuals might not aggress against kin has also been advanced by Coyne and Thomas (2008), “According to evolutionary theory … psychopaths would be more likely to use such (aggressive) behavior against strangers, associates, or colleagues, as compared with romantic relationships or family members (p. 1113).” We assert: (1) the finding that violent psychopathic offenders are more frequently imprisoned for stranger victimization does not necessarily imply they do not also victimize kin in a way that would “impair fitness,” (2) most psychopathic individuals are not violent and so non-violent victimization is perhaps more important in this group, and (3) even if present, lack of violent harm does not equate to nepotistic help.

We applaud these authors for raising the issue of the treatment of relatives by psychopathic individuals as it is commonly asserted that offenders do better when they have supportive family ties (Andrews and Bonta, 2003). This assertion has caused mental health and criminal justice professionals to encourage family ties (Rotgers and Maniacci, 2005). There is little data regarding the impact of offenders on the family members who are asked to act as supports; if psychopathy is associated with harm to family members, it may not be ethical to encourage family ties. It is difficult to study the impact of psychopathy on the family because of ethical constraints on research involving prisoners and lack of access to psychopathic individuals in the community (Widom, 1977). A review of Cleckley's cases (1964), surveys of people who claim relationships with psychopathic individuals (Leedom and Andersen, 2011), and qualitative analyses of memoirs written by the wives and adult sons and daughters of highly psychopathic individuals (Leedom et al., 2012, submitted) indicate that family members report considerable harm including psychological, emotional, financial and physical abuse, and exploitation. Sons and daughters also report being encouraged to engage in antisocial behaviors. These studies do not however provide data about the rate of harm because the subjects are self-selected for the occurrence of harm. Controlled studies comparing psychopathic individuals in the community to matched controls are needed to prove hypotheses regarding harm or aid to family members. Interestingly, the memoire studies do suggest that psychopathic individuals will aid family members if they perceive a benefit to themselves for doing so (Leedom et al., 2012, submitted). Also reported was *nepotistic assistance to highly psychopathic individuals by less psychopathic kin*. Psychopathy may persist in human populations in part because of kin support *to* (not from) psychopathic individuals.

## Is psychopathy a mental disorder?

The paper cites Wakefield (1992),

I propose a hybrid account of disorder as harmful dysfunction, wherein dysfunction is a scientific and factual term based in evolutionary biology that refers to the failure of an internal mechanism to perform a natural function for which it was designed, and harmful is a value term referring to the consequences that occur to the person because of the dysfunction and are deemed negative by sociocultural standards (p. 374).

According to one prevailing theory of psychopathy, the *integrated emotions systems (IES) model* (Blair et al., 2005), psychopathic individuals are impaired in their processing of interpersonal cues associated with fear and distress. Consequently, psychopathic individuals fail to heed cues that would otherwise lead them to inhibit aggressive behavior. This theory points to *the failure of an internal mechanism to perform a natural function for which it was designed*. That psychopathy is associated with disastrous consequences for individuals and society was not disputed by the authors. Any “benefit” of psychopathy according to the paper is in perpetuation of genes only. However, that psychopathic individuals might contribute to the gene pool, has no bearing on the definition of harm as conceptualized by Wakefield.

The authors also suggest “psychopathy is neither co-morbid nor associated with the neurodevelopmental perturbations characteristic of other serious mental illnesses, such as psychosis (p. 1).” We disagree, as there is significant co-morbidity between paranoid personality disorder and psychopathy (Blackburn and Maybury, 1985; Blackburn, 1998; Fullam and Dolan, 2006; McGregor et al., 2012). We point to Eysenck's concept of psychoticism, which links psychopathy to a tendency to become psychotic (Eysenck and Eysenck, 1977; Corr, 2010). Genetic studies have even identified potential genes linking psychoticism to schizophrenia (Suchankova et al., 2012). In the aforementioned memoire study, psychoticism was a ubiquitous finding in husbands and parents with high PCL-R scores. DSM 5 includes a measure of psychoticism in personality disorder assessments (American Psychiatric Association, 2012). Hence future research will clarify the relationship between psychoticism and psychopathy. We conclude that the contention that psychopathy does not meet Wakefield's definition of disorder was not proven by the author's arguments.

## Behavioral systems and psychopathy

According to the behavioral systems perspective (BSP) (Johnson et al., 2012; Leedom, submitted) there are four social behavioral systems that have been subjected to adaptive selection in primate evolution: the attachment, caregiving, dominance, and sexual systems. In the BSP, psychopathy is associated with excessive sexual responses, lack of caregiving, and aberrant dominance responses. The lack of caregiving termed lack of “love” in the paper could be another *failed internal mechanism* in psychopathy. The BSP hypothesizes that violent behavior and the interpersonal symptoms of psychopathy are a consequence of aberrant development of the dominance system (Johnson et al., 2012; Leedom, submitted).

Rather than being “an adaptation” psychopathy may represent a spandrel—a syndrome that arises as a consequence of other features (Buss et al., 1998; Cuzzillo, 1999). We hypothesize that psychopathy may arise in part due to selection for social dominance (and possibly mating effort) and assert that it is not psychopathy but dominance (combined with mating effort) that confers adaptive advantage. Viewed as a “spandrel,” psychopathy is no more an “adaptation” than are anxiety disorders and depression, which represent diminished rather than enhanced dominance system function (Johnson et al., 2012). Recent research by Hawley (2002) on the development of the dominance system supports the BSP of psychopathy, and also suggests that “caregiving” or “prosocial” responses may be dominance behavior as these are used instrumentally by individuals of all ages to gain power. Hence, “nepotism” and other “prosocial behavior” displayed by psychopathic individuals is likely a function of the dominance as opposed to the caregiving system. New assessment instruments are needed to determine the presence of specific motives and the function of behavioral systems in psychopathic individuals as related to prosocial and antisocial behavior (Leedom et al., 2012).

## Conclusion

We strongly dispute the assertion that psychopathy is not a disorder. Psychopathy is a mental disorder according to both the Wakefield definition cited in this study and American Psychiatric Association criteria (American Psychiatric Association, 2000). More studies of the harm done to family members by psychopathic individuals are needed. Psychopathy may persist because it represents a dominance-related spandrel and because of nepotistic help to, not from, psychopathic individuals.
